# Accuracy and precision of small saccades

**DOI:** 10.1038/s41598-020-72432-6

**Published:** 2020-09-30

**Authors:** Martina Poletti, Janis Intoy, Michele Rucci

**Affiliations:** 1 Department of Brain and Cognitive Sciences, University of Rochester, Rochester, NY 14627 USA; 2 Department of Neuroscience, University of Rochester, Rochester, NY 14627 USA; 3 Center for Visual Science, University of Rochester, Rochester, NY 14627 USA; 4 Graduate Program for Neuroscience, Boston University, Boston, MA 02215 USA

**Keywords:** Motor control, Saccades

## Abstract

Despite recent advances on the mechanisms and purposes of fine oculomotor behavior, a rigorous assessment of the precision and accuracy of the smallest saccades is still lacking. Yet knowledge of how effectively these movements shift gaze is necessary for understanding their functions and is helpful in further elucidating their motor underpinnings. Using a combination of high-resolution eye-tracking and gaze-contingent control, here we examined the accuracy and precision of saccades aimed toward targets ranging from $7^\prime$ to $80^\prime$ eccentricity. We show that even small saccades of just 14–$20^\prime$ are very effective in centering the stimulus on the retina. Furthermore, we show that for a target at any given eccentricity, the probability of eliciting a saccade depends on its efficacy in reducing the foveal offset. The pattern of results reported here is consistent with current knowledge on the motor mechanisms of microsaccade production.

## Introduction

Humans use rapid eye movements, known as saccades, to place objects of interest on the foveola, the region of the retina where acuity is highest. Even after an object is foveated, small saccades—commonly referred to as microsaccades—continue to occur, shifting the retinal projection of the stimulus within the foveola itself.

It is now known that microsaccades serve recentering functions similar to those of larger saccades, by finely positioning the stimulus on the retina in high-acuity tasks^1,2^. Multiple observations suggest that these movements should be relatively accurate^3–5^. Furthermore, recent evidence indicates that they also need to be precise, as performance in high-acuity tasks drops rapidly with distance from the preferred fixation locus within the foveola^2^. However, whereas the performance of saccades has been extensively characterized^6–11^, a rigorous analysis of the accuracy (average landing error) and precision (landing variability) of microsaccades faces experimental challenges. Yet, evaluation of accuracy and precision is essential to determine how humans use these movements and to further elucidate their motor mechanisms.

**Figure 1:**
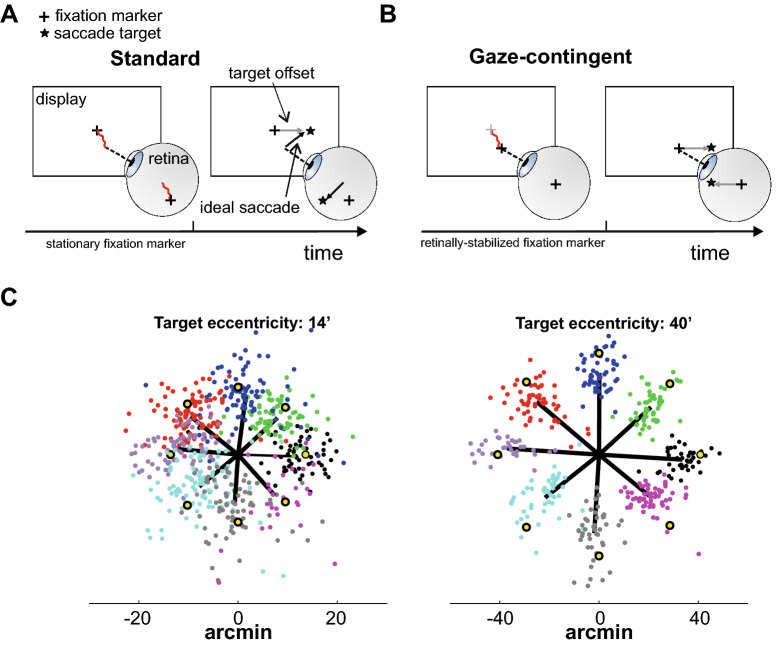
Experimental paradigm. (**A**) Saccade performance is normally evaluated relative to the displacement between the target (star) and the fixation marker (cross) on the display (gray arrow). This approach does not work well with small saccades, because the normal wandering of the eye (eye drift; red line) displaces the stimulus on the retina by an amount comparable to the saccade itself, so that the resulting offset (black arrow) deviates from the target-fixation vector. (**B**) In this study, the eye displacement occurring before the target’s onset was eliminated via retinal stabilization. The fixation marker moved with the eye so to remain immobile at the center of the preferred retinal locus of fixation (the center of gaze), and the target appeared at a desired eccentricity from this point. To maintain the normal retinal motion present during saccade preparation, retinal stabilization was turned off at the appearance of the target. (**C**) Landing positions of saccades executed toward targets at $14^\prime$ and $40^\prime$ distance from the center of gaze. Each data point represents a saccade vector color-coded according to the target (yellow dots). Black lines are the average saccades to each target. Data are from one participant.

Assessing the performance of very small eye movements in humans is experimentally challenging for two primary reasons. First, the spatial accuracy necessary for this purpose goes beyond that provided by commonly used eye-tracking methods, such as video-based eye-trackers^12–15^. Unlike studies with non-human primates, which have traditionally relied on more invasive approaches, standard measurements in humans yield relatively large regions of uncertainty in the localization of the line of sight, which can be as large as the small gaze shifts one wants to study. This severely limits measurement of saccade landing position. Second, the traditional procedures used to examine larger saccades do not easily extend to small eye movements. These paradigms typically compare saccade characteristics to the vector between the positions of the fixation marker and the saccade target on the display^6–11^ (see Fig. 1). These approaches work well for relatively large saccades, for which one can ignore the physiological instability of fixation. However, incessant eye movements during fixation introduce offsets that are comparable to the amplitude of microsaccades and need to be taken into account in examining behavioral performance.

Standard paradigms also carry deeper consequences. The discrepancy between the ideal saccade and the retinal vector subtended by the saccade target and the fixation marker may actually interfere with the planning and execution of the saccade, affecting the very quantities we want to measure. Part of this discrepancy is natural, as the eyes always drift *during* saccade preparation in normal viewing. However, in the laboratory, it is unnaturally amplified by the eye movements that occur *before* the onset of the saccade target, which may bring the line of sight far from the fixation marker. Furthermore, the explicit request to maintain fixation draws attention to the fixation marker, possibly enhancing the influence of the target-marker vector at the expenses of saccade execution.

To circumvent these problems, in this study we relied on high-resolution eye-tracking coupled with gaze-contingent control. We used a custom calibration that enables high accuracy in gaze localization^2,16,17^, and modified the standard procedure previously used for larger saccades to reduce the presence of conflicting cues. Following this approach, we examined the accuracy and precision of saccades smaller than $80^\prime$. We show that these small gaze shifts are highly effective in reorienting the line of sight toward the stimulus.

## Results

Ample variability exists in the literature in the use of the term microsaccade^12^. To avoid possible confusion, in the following, we will refer to all saccadic eye movements collectively as small saccades, without attempting further distinction.

**Figure 2:**
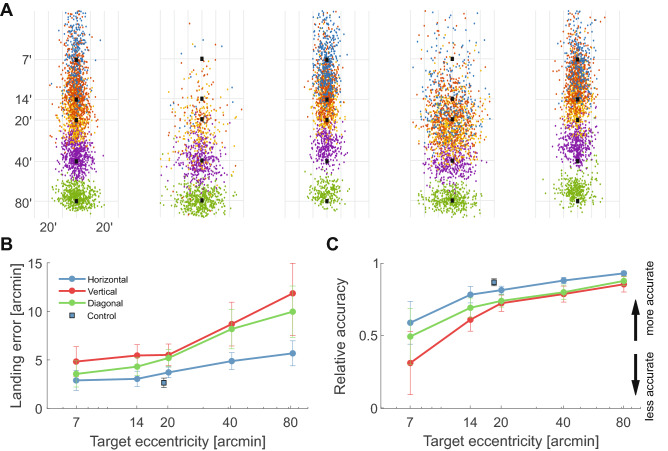
Saccade accuracy. (**A**) Saccade landing positions for targets at various distances ($7^\prime$-$80^\prime$; different colors). Each panel shows data for one individual subject, with each dot representing one saccade. Black squares mark target locations. Data from different target angles are realigned along the vertical axis for better visualization. (**B**) Mean landing error ($S_E$). The average distance between saccade landing and target’s position is plotted as a function of target’s eccentricity ($T_E$). Error bars represent SEM. (**C**) Index of relative accuracy, defined as $1-S_E/T_E$. Error bars represent SEM. Panels *B* and *C* also show results obtained in a control experiment (squares), which relied on precise gaze localization to replicate the standard approach used for larger saccades (Fig. 1A). In this experiment, subjects (*N*=6) performed saccades toward targets at 20$^\prime$ from an unstabilized fixation marker, but only trials with accurate fixation were selected for data analysis.

Figure 1 describes the experimental procedure of this study, emphasizing its difference relative to the standard approaches previously used in the literature to examine larger saccades. Unlike previous methods (Fig. 1A), the fixation marker was here stabilized on the retina: it moved on the display, according to the observer’s eye movements, to remain at the center of the preferred retinal locus of fixation (Fig. 1B). Once steady fixation was attained, a saccade target appeared at the desired location and retinal stabilization was deactivated, so that the observer could normally shift gaze toward the target. This approach minimizes discrepancy between the ideal saccade and the target-marker vector, while allowing for normal retinal image motion *during* saccade preparation. In this study, saccade targets appeared at various positions relative to the center of gaze so to cover a range of nearby distances (from 7$^\prime$ up to 80$^\prime$) in 8 possible directions spanning 360$^\circ$. Subjects were capable of executing the task without apparent effort, consistently redirecting their gaze toward the target, as requested (see example in Fig. 1C and individual partecipant’s data in Fig. 2A).

We first focus on saccade accuracy, *i.e.*, the degree by which saccades tend to land on target. To this end, we estimated the landing error, defined as the Euclidean distance between the landing position of the saccade and the target location. As expected, the landing error increases with the target distance (Fig. 2B). It more than doubled within the range of examined distances, increasing from an average error of $4^\prime \pm 3^\prime$ for saccade aimed at targets just $7^\prime$ away to $9^\prime \pm 5^\prime$ for targets at $80^\prime$ (Fig. 2B).

Landing errors are not just a function of distance, they also depend on the angular eccentricity of the target. Similarly to what was already reported for much larger saccades^7^, gaze shifts were considerably more accurate along the horizontal axis than along the vertical and diagonal axes. Vertical saccades were on average 40% less accurate than horizontal ones, a percentage that remained approximately constant over the range of examined distances. Furthermore, consistent with previous observations^18^, saccades with upwards components were on average more accurate than those with downwards components (a 12% difference; $p=0.019$, paired t-test).

How efficient are saccades in redirecting gaze? A small landing error does not necessarily imply that a saccade is efficient in re-centering the stimulus on the preferred retinal locus. Given the amplitudes of the saccades considered here, landing errors will always be small, even for saccades that end up increasing the foveation error by landing farther away from the target. A relative measure of saccade accuracy can be obtained by normalizing the landing error $S_E$ by the target’s eccentricity $T_E$.

Figure 2C shows the index of relative accuracy, defined as $1 - S_E/T_E$. With this metrics, a saccade that perfectly lands on the stimulus yields a unit index, whereas a saccade that increases the distance of the target from the center of gaze gives a negative value. The data show that saccades made toward targets at $7^\prime$ eccentricity are already quite efficient in reducing the retinal error. Their relative accuracy index was on average $0.5\pm 0.4$, meaning that they reduced the pre-saccadic error by approximately half the distance. Small increments in saccade amplitudes led to considerably higher index values, so that each individual subject exhibited higher relative accuracy at $14^\prime$ than that at $7^\prime$ ($p<0.05$; nonparametric bootstrap). Saccades made toward targets at $14^\prime$ reduced the retinal error by approximately $70\%$. Slightly larger saccades were ever more efficient, particularly on the horizontal axis, where on average they reduced the retinal offset by almost 90%. These data show that small saccades tend to be remarkably accurate in recentering gaze. In all tested observers, saccades toward targets at just $20^\prime$ reduced the retinal error by at least 60% ($p<0.01$, nonparametric bootstrap).

**Figure 3:**
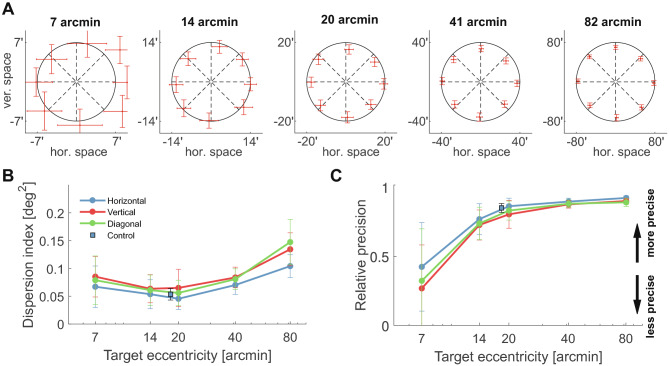
Saccade precision. (**A**) Average dispersion in saccade landing for the same targets as in Fig. 2. The error bars now represent the mean standard deviation of landing error, evaluated for each individual observers and averaged across subjects. Error bars are centered at the mean landing position. (**B**) Dispersion index (DI), expressed as the area of the $68^{\mathrm{th}}$ percentile ellipsoid in the distribution of landing positions. (**C**) Index of relative precision, defined as $1-DI/T_E$, where $T_E$ is the target eccentricity. In both *B* and *C*, error bars represent SEM. As in Fig. 2, squares refer to data collected in a control experiment with unstabilized fixation marker.

Even highly accurate saccades may not be beneficial if their precision is poor, *i.e.,* their landing positions vary extensively. We, therefore, examined the dispersion in saccade landing for every target position (Fig. 3A). Like accuracy, also precision varied with the amplitude and direction of saccades. The mean deviation in saccade landing remained approximately constant as the eccentricity of the target ranged from $7^\prime$ to $20^\prime$ and increased for saccades made toward targets at larger eccentricities. Irrespective of the target’s distance, saccades on the horizontal axis were always the most precise, followed by diagonal and vertical saccades. As for the accuracy, saccades that shifted gaze into the upper visual field were also more precise than those moving toward the lower field, with an average reduction in landing dispersion of 23% ($p=0.0004$; paired t-test).

These results are summarized in Fig. 3B by means of the dispersion index (DI), the area contained within one standard deviation from the average landing position. This area is the direct equivalent in two dimensions of the variance for a one-dimensional variable^19^. On average across all directions, the dispersion index doubled as the target’s distance increased by over an order of magnitude. It ranged from a minimum of $0.06\pm 0.05\,\hbox {deg}^2$ at $14^\prime$ eccentricity to $0.13\pm 0.07\,\hbox {deg}^2$ at $80^\prime$ ($p< 0.01$, two-tailed paired t-test), an increment statistically significant for each individual observer ($p<0.001$; nonparametric bootstrap). Thus, saccades to targets closer to the center of gaze were actually more precise than saccades to targets located further away.

Again, a smaller dispersion does not necessarily imply a more efficient movement. In Fig. 3C, the same data of Fig. 3B are replotted now normalized by the eccentricity of the target, so to examine the variability in saccade landing relative to its amplitude. The relative precision of saccades improved sharply for targets at $14^\prime$ and $20^\prime$ and continued to improve, although less markedly, for targets at greater eccentricities. In every subject, the change in relative precision was larger between $20^\prime$ and $7^\prime$ than between $80^\prime$ and $20^\prime$ ($p<0.001$; nonparametric bootstrap). Thus, in relative terms, smaller saccades were actually less precise in shifting the gaze, as the variability in landing position was as large as the saccade amplitude (see also Fig. 3A).

Our main conclusion that saccades as small as $20^\prime$ are accurate and precise is further supported by the results of a a control experiment, in which we used the traditional paradigm used to study larger saccades, but relied on our capability for accurately localizing gaze to properly select trials. In this control experiment, as in previous experiments in the literature, we: (a) presented a single fixation marker (a $2^\prime$ dot); and (b) presented the saccade target at a fixed distance ($20^\prime$) relative to the fixation marker, rather than the center of gaze. As mentioned above, because of the incessant wandering of gaze, this procedure normally introduces a discrepancy between the ideal saccade and the vector subtended between the saccade and the fixation marker. However, we minimized this problem by using our accurate localization of the line of sight and only selecting the trials in which gaze remained within a distance of $5^\prime$ from the fixation marker. As shown in Figs. 2B and 3B, accuracy and precision measured in this control were highly similar to those measured in our main experiment ($p=0.2$ and $p=0.7$ for accuracy and precision, respectively; unpaired *t*-test).

**Figure 4:**
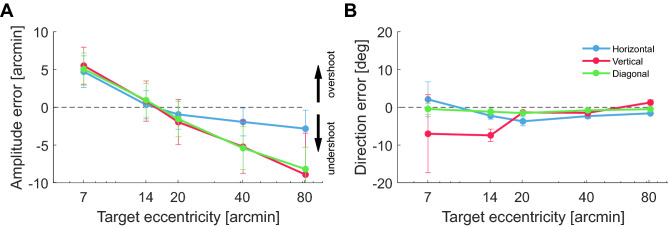
Accuracy in amplitude and direction. Average error in (**A**) amplitude and (**B**) direction for saccades aimed toward targets at various eccentricities. Error bars represent SEM.

**Figure 5:**
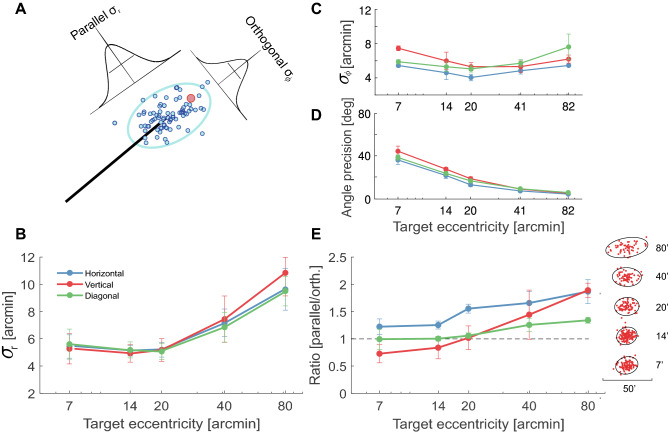
Precision in amplitude and angle. (**A**) Decomposition of variability on the two axes parallel and orthogonal to the saccade, $\sigma _{r}$ and $\sigma _{\phi }$. The black line represents the average saccade trajectory, the red circle gives the position of the target, and the blue dots are the landing positions of individual saccades. The 95% confidence ellipse is also shown. (**B**–**E**) Values of $\sigma _r$ (**B**), $\sigma _{\phi }$ (**C**), angular precision (**D**) and scatter ratio $\sigma _r/\sigma _{\phi }$ (**E**) as a function of target eccentricity. Error bars represent SEM. The three lines refer to saccades in different directions. In (**E**), the dashed line represents a spherical distribution; the scatter plots are the 2D landing distributions and their 95% confidence ellipses for one subject.

The measurements of accuracy and precision in Figs. 2 and 3 were obtained using the standard Cartesian reference frame defined by horizontal and vertical axes. However, it is also informative to examine the degree of control in angle and amplitude. To this end, we reanalyzed experimental data in polar coordinates (Figs. 4 and 5).

Figure 4A shows the average amplitude error, defined as the difference between saccade amplitude and target eccentricity. The overall accuracy in saccade amplitude changed across the eccentricity range considered in our experiments: from $5^\prime$ to -$7^\prime$ for the closest and farthest targets, respectively. Small saccades toward targets at $14^\prime$ eccentricity were, on average across subjects, the most accurate in terms of amplitude, with a mean error of just $1^\prime$. In agreement with previous observations ^20^, whereas saccades aimed at closer stimuli overshot the target, those toward stimuli farther away exhibited a consistent undershoot, which was particularly pronounced for vertical and diagonal saccades. Every individual subject exhibited this bias toward shorter saccades with increasing target eccentricity: in all observers, the amplitude-eccentricity difference was lower for the farthest targets ($80^\prime$) than for targets at eccentricities $\le 20^\prime$ ( $p<0.001$, nonparametric bootstrap).

A different pattern of results emerged for the directional accuracy (Fig. 4B). In contrast to the amplitude, the error in saccade angle remained approximately constant across the range of examined eccentricities. The mean angular error, defined as the difference in angle—averaged across all saccade directions—between the saccade vector and the vector connecting the target to the fixation marker, was approximately $1.2^\circ \pm 6^\circ$ for the targets closest to the center of gaze ($7^\prime$ and $14^\prime$ eccentricities) and -$0.4^\circ \pm 0.4^\circ$ for targets at $80^\prime$. This small trend led to a significant difference in angular accuracy between the smallest (7-$14^\prime$) and largest (40-$80^\prime$) saccades considered ($p=0.0035$; two-tailed paired K-test). Note, however, that most of this error came from vertical saccades, which were considerably less accurate for very nearby targets and on par with the others for more distant targets. In every participant, the angular accuracy of saccades at $20^\prime$ was virtually identical and statistically indistinguishable from that of larger saccades. In sum, the smaller the saccade, the more likely it is to overshoot the target, but the directional accuracy remains approximately the same, except for vertical saccades.

We also examined the precision of saccade landing along the axes parallel and orthogonal to the saccade vector (Fig. 5A). For saccades larger than $2^\circ$, it has been previously reported that the scatter of saccade landing on both axes grows with saccade amplitude^8^. Our data show a different behavior for small saccades. The dispersion along the parallel axis remained constant for saccades aimed at targets in the range $7^\prime$–$20^\prime$ and increased by approximately 80% for more distant targets ($p=0.04$, two-tailed paired t-test; Fig. 5B). This decrement in landing precision was statistically significant in each subject individually ($p<0.001$; nonparametric bootstrap). In contrast, on the orthogonal axis, the dispersion of saccade landing changed little with saccade amplitude (Fig. 5C). This implied that, for every individual observer, the angular dispersion—the standard deviation in saccade angle—decreased as the amplitude of the saccade increased (Fig. 5D; $p<0.01$, nonparametric bootstrap). Again, differences between saccades with upwards and downwards vertical components were visible: a 16% increase in amplitude precision was observed for saccades toward targets at $40^\prime$ in the upper visual field ($p=0.008$; paired t-test). Furthermore, with targets at $20^\prime$ and $40^\prime$, upward saccades also exhibited, respectively, 11% and 18% improvements in direction precision relative to downward saccades ($p=0.009$ and $p=0.02$; paired t-tests).

For saccades larger than those considered here (amplitude $>2^\circ$), previous studies have also reported higher precision on the orthogonal axis than on the axis parallel to the saccade direction^8,11^. This yields a characteristic elliptical distribution of saccade landing, in which the longer axis, the one aligned with the saccade, possesses approximately double dispersion than the other^8^. In contrast, in our data, saccades elicited in response to targets at $14-20^\prime$ exhibited approximately equal dispersion on the two axes, so that their landing distributions were close to circular (Fig. 5E). For all individual subjects, the scatter ratio was significantly smaller than 1.5 for targets at eccentricities $\le 20^\prime$ ($p<0.05$, nonparametric bootstrap). For targets at larger eccentricities, the distributions of saccade landings became gradually more elliptical, stretching along the saccade direction. This change in the ratio between the standard deviations on the two axes was present in all participants and reached statistical significance in four of them ($p<0.01$, nonparametric bootstrap test). On average across observers, the scatter ratio approximately doubled over the range of examined eccentricities, suggesting that it has already reached its plateau at approximately $80^\prime$.

**Figure 6:**
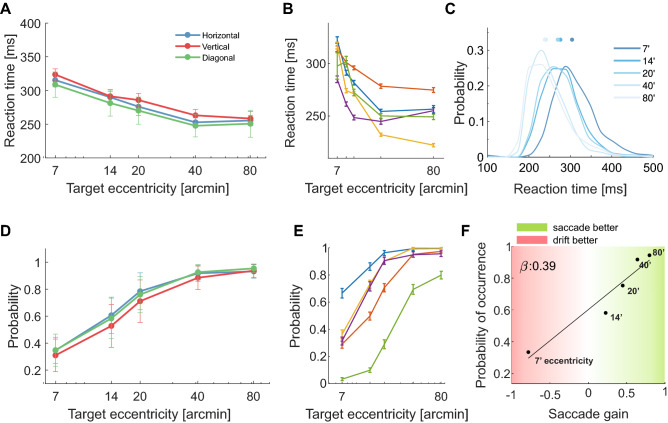
Saccade latencies and probability of occurrence (**A**–**C**) Saccade reaction times for targets at various eccentricities. (**A**) Averages across observers for saccades in the three directions. Data points represent medians (circles) and SEM (error bars). (**B**) Individual subject data (all directions have been grouped together). Error bars represent SEM. (**C**) Mean reaction times distributions. (**D**, **E**) Probability of executing a saccade as a function of the distance of the target. Both averages across observers (**D**) and the individual subject data (**E**) are shown. Error bars represent SEM in *D* and bootstrapped confidence intervals in (**E**). (**F**) Probability of executing a saccade as a function of its gain. The saccade gain is the normalized difference between the mean retinal errors in the trials in which target onset did and did not elicit a saccade. Negative/positive gains indicate conditions in which saccades result in larger errors than maintaining fixation via ocular drift. Each point represents the average probability of saccade occurrence and the average gain at a given target eccentricity. The solid line (slope $\beta$) is the linear best fit to the data.

Saccades of different amplitudes not only differed in accuracy and precision, they also possessed different reaction times. Consistent with previous studies^21,22^, all subjects were noticeably faster in eliciting saccades when targets were at larger eccentricities ($p<0.001$, nonparametric bootstrap; Fig. 6B). On average, reaction times decreased from $314\pm 18$ ms with targets at $7^\prime$ to $254\pm 18$ ms with targets at $80^\prime$ ($p=0.039$, two-tailed paired t-test), an effect present for all saccades independent of their directions (Fig. 6A). It led to distributions of reaction times that resembled Poisson distributions with different means (Fig. 6C). Interestingly, in every individual observer, smaller saccades were not only associated with longer reaction times, they also possessed a lower probability of occurrence ($p<0.001$, nonparametric bootstrap; Fig. 6E), a finding consistent with the idea of a “dead zone” around the line of sight in which targets do not trigger saccadic movements^23,24^. On average, observers failed to execute a saccade in 70% of the trials with targets at $7^\prime$ distance. This percentage of trials without a behavioral response diminished as the distance of the target increased. It was approximately 50% with targets at $14^\prime$ and dropped to just 5% with targets at $80^\prime$ (Fig. 6D).

In the trials in which the onset of the target failed to elicit a saccade, the eye continued to move because of ocular drift, the incessant intersaccadic motion of the eye. This behavior may actually reflect a good fixation strategy, as several studies have indicated that, like microsaccades, ocular drift is also controlled^25–28^. For targets located only a few arcminutes away from the center of gaze, it may, in fact, be more convenient to maintain fixation rather than perform a saccade, since the latter may end up with comparable or even larger retinal error. That is, a possible explanation for the varying degree of behavioral responsiveness is that the probability of executing a saccade depends on how effectively the saccade reduces the retinal error relative to ocular drift. This hypothesis specifically predicts that the probability of saccade occurrence should depend on the difference between saccade accuracy and the accuracy of ocular drift.

To assess if this is the case, for each target’s distance, we compared the retinal errors resulting from saccades and eye drifts. The horizontal axis in Fig. 6F represents the saccade gain, the relative difference (normalized by target distance) between the average retinal errors across the trials in which the stimulus did and did not elicit a saccade. When the gain is close to zero, performing a saccade and maintaining fixation via ocular drift bear comparable consequences. Negative gains represent conditions in which drift is more beneficial than a saccade, whereas the opposite occurs for positive gains. We would expect subjects to suppress their saccades when aimed toward targets that yield negative saccade gains and to refrain from doing so when the saccade gains are positive. Consistent with these predictions, the data in Fig. 6F show a monotonic, almost linear relationship between saccade probability and the saccade gain: the greater the average benefit of the saccade, the more likely is a target at a given eccentricity to elicit a saccade. The probability of performing a saccade increased with saccade gain in all individual subjects, so that gains larger than 0.5 always yielded higher saccade probability than lower gains ($p<0.001$, nonparametric bootstrap). Thus, the relative difference between saccade and drift accuracies is a reliable predictor of saccade occurrence.

## Discussion

Small saccades, often referred to as microsaccades, occur frequently during normal vision. It has long been known that these movements can be voluntarily executed^3,4^, and recent research has shown that they tend to re-center the preferred retinal locus on nearby objects of interest even if the attended stimulus is already within the foveola^1,2,33^. Yet little is known about their degree of accuracy and precision, as estimating these quantities presents multiple challenges, both technical, related to the need for finely localizing gaze, and procedural, concerning the design of methods that minimally interfere with oculomotor measurements. Here, we leveraged on the advantages of gaze-contingent control to overcome these challenges. Our results show that, while important differences exist, saccades as small as $14^\prime$-$20^\prime$ possess accuracy and precision comparable to those of much larger saccades.

More specifically, the results of our experiments indicate that, as the saccade amplitude increases by over an order of magnitude (from $7^\prime$ to $80^\prime$), the foveation error and the landing variability change by a relatively small fraction, particularly for horizontal saccades. In absolute terms, both accuracy and precision remain approximately constant up to $20^\prime$ and decrease by a factor of 2 between $20^\prime$ and $80^\prime$. In relative terms, with reference to the target’s distance, these data imply that only the smallest saccades, those elicited by targets at $7^\prime$, carry considerably lower accuracy and precision than the others. The lower accuracy of these minute gaze shifts originates primarily from amplitude errors, which transition from undershooting to overshooting the target at around $20^\prime$ eccentricity. Their lower precision stems primarily from an almost constant landing dispersion on the axis orthogonal to saccade direction, which yields larger angular variability for smaller amplitudes. These imperfections may not be critical for visual functions. Fine pattern vision appears to be uniform within a region of at least $5^\prime$ around the preferred retinal locus^2^, and angular imprecision has minimal repercussion on the landing error of very small movements.

These results were possible thanks to a combination of advanced eye-tracking techniques. First, we relied on Dual Purkinje imaging for high-resolution eye-tracking. This technique has been extensively tested over the course of several decades and is known provide a resolution of approximately $1^\prime$^16,34^. Second, to improve gaze localization, we used a gaze-contingent calibration procedure in which subjects iteratively refined the estimated line of sight by correcting its offsets. We have previously shown that this procedure reduces the region of uncertainty by approximately one order of magnitude^2,17^. Third, we used a gaze-contingent paradigm to minimize motor and visual discrepancies, while allowing for the normal retinal image motion that occurs during saccade planning. The fixational marker was stabilized on the retina to remain centered on the preferred fixation locus before the onset of the saccade target. This ensured that, on the retina, the target’s displacements relative to the center of gaze and the fixation marker—the intended position of gaze—were identical at the moment of target onset and only varied during the period of saccade preparation, as it occurs naturally.

Most previous studies on the subject date back several decades, to a period in which high-resolution oculomotor measurements were achieved via more invasive methods, and absolute localization of the line of sight was difficult^3–5,22^. These older studies primarily focused on the saccades triggered by the sudden shift of a fixation marker. They reached somewhat conflicting conclusions in terms of the efficacy of microsaccades, with some reporting a residual retinal error of $\sim 50\%$^4,37^ and others settling on considerably lower errors^5,22^. However, since these studies focused on the amplitude of the triggered saccade, not its actual landing position, their results are only indirectly related to saccade accuracy and precision.

Older studies also lacked methods like our gaze-contingent calibration for accurately localizing the line of sight. As a consequence, they could not fine-tune the position of the saccadic target to compensate for fixational offsets. Under these circumstances, during the physiological instability of visual fixation, the more the line of sight moved away from the fixation marker, the more the ideal saccade differed from the reference, the vector subtended by the saccade target and the marker. This raises the possibility that different degrees of fixational instability in the pools of tested subjects could have contributed to the discrepancies in previous reports. Our results are not affected by this problem. Retinal stabilization and accurate gaze localization ensured that the ideal saccade always coincided with the target-marker vector, eliminating possible confounds from fixational instability.

In our experiments, the stimulus was designed to facilitate fixation, and trials with unusually large fixational instability were discarded to ensure high-quality retinal stabilization. One may wonder whether these precautions led to an unnaturally high stability of visual fixation, which could have, in turn, inflated measurements of saccade accuracy and precision. Two considerations indicate, however, that this is unlikely. First, the percentage of trials that was discarded because of inaccurate fixation was small: less than 5% of the total number of trials for each individual subjects. Second, fixational instability was comparable to that measured in more natural tasks. For example, on both axes, the average dispersions of gaze in our experiments ($5.1^\prime \pm 1.6^\prime$ and $5.5^\prime \pm 1.7^\prime$ on the horizontal and vertical axes, respectively) were almost identical to those measured in a larger pool of subjects engaged in judging the expression of faces seen from a distance, a task that naturally elicits microsaccades toward the most task-relevant features^33^ (horizontal: $5.1^\prime \pm 2.5^\prime$; vertical: $5.8^\prime \pm 4.9^\prime$; $p=0.5$ and 0.3, respectively; Wilcoxon rank sum test; $N=24$). Thus, our procedure does not appear to alter the fixational behavior preceding saccades.

It is worth pointing out that the unit of observation of our study is the experimental trial and that our conclusions are based on large number of measurements for each subject. This enabled estimation of confidence intervals for each individual observer. All the main effects reported in this study were significant in each participant. Under standard statistical assumptions, this guarantees that the findings generalize to the broader population, as we can confidently conclude ($p<0.05$) that the effects will be present in the majority of the population^38^. A few observations also suggest that our observers are well representative of the performance of young, healthy adults. Within our pool of subjects, relatively little individual variability was measured in both saccade accuracy and precision. In terms of accuracy, the standard deviation of the landing error, averaged across subjects, was only $5^\prime$ at its peak, the condition with the largest saccades (those toward targets at $80^\prime$). In terms of precision, the standard deviation of the dispersion index was approximately $0.06\,\hbox {deg}^2$. Thus, all subjects performed saccades with highly similar characteristics. In addition, we observed almost identical accuracy and precision in a control experiment conducted with different subjects, in which stimuli were displayed at fixed position of the monitor, rather than in a gaze-contingent manner. This experiment replicated the standard procedure used to study larger saccades^11^, but we used our capability for finely localizing the line of sight to select trials with little discrepancy between the target offset vector and the ideal saccade (see Fig. 1A).

Our findings on the latency and probability of occurrence of small saccades are consistent with previous reports. In keeping with our results, it has already been reported that the latency-eccentricity function is bowl-shaped and that latency increases for saccades smaller than $1^\circ$^21^, reaching more than 300 ms^22,39^. Such long reaction times are very rarely observed with saccades larger than $1^\circ$^40^. Furthermore, several previous studies have noticed that minute shifts in the fixation marker of the order a few arcminutes may occasionally fail to trigger saccades, whereas slightly larger shifts do not^4^. Our study adds to this previous literature by noticing that small saccades differ from larger saccades in other dimensions besides latency, including an amplitude overshoot rather than an undershoot, a lower angular precision, and a more circular distribution of landing positions. Moreover, it proposes a computational mechanism—the comparison between saccade and drift accuracy—that accounts for the observed changes in the probability of saccade occurrence with saccade amplitude.

**Figure 7:**
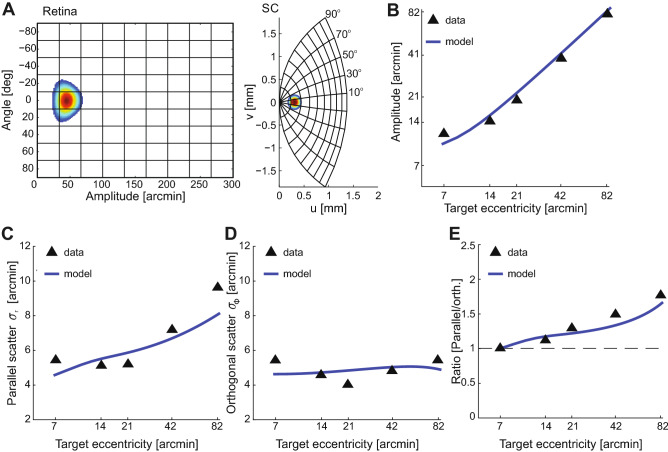
Collicular representation predicts microsaccade characteristics. (**A**) Mapping of visual space from the retina to the superior colliculus. The heatmap shows the representation of a single visuomotor response field in both coordinate systems. This transformation well predicts several characteristics of small saccades. (**B**–**E**) Comparison of experimental data for horizontal saccades (triangles) to saccade metrics derived from a neural population model that sums activity in a log-polar map (solid lines; see text for details) for targets at various eccentricities on the horizontal meridian. (**B**) Amplitude accuracy. (**C**) Amplitude precision. (**D**) Precision on the axis orthogonal to saccade direction. (**E**) Scatter ratio.

Our results also fit well with current knowledge regarding the neural substrate underlying microsaccade production. Recent research has pointed at an involvement of the rostral region of the superior colliculus in the generation of microsaccades^41,42^. It is well established that the deep layers of the superior colliculus contain a motor map of space organized in polar coordinates with a roughly logarithmic representation of saccadic amplitude^43–45^ (Fig. 7A). This model well predicts saccade accuracy and precision^8^. Fig. 7 shows that, with minimal changes, this model also fits well our experimental measurements, including the saccadic overshoot for the smallest saccades (Fig. 7B), the decrement in precision with increasing saccade amplitude along the axis parallel to the movement (Fig. 7C), and the relatively constant precision on the orthogonal axis (Fig. 7D). Relative to its original formulation, the model was here modified in two ways. First, it allowed for an expanded representation of the fovea, as measured by recent studies^46^. Second, it allowed for activity fields that are not radially symmetric, consistent with an earlier observation^8^. These modifications yield more comparable landing dispersion along the two axes for the smallest saccades (Fig. 7E). Thus, our results appear compatible with the notion of a very fine representation of foveal space in the superior colliculus ^46^.

Furthermore, the idea that microsaccade production depends on an activity imbalance between the two colliculi^42^ and the fact that rostral neurons are characterized by a higher baseline activity and higher variability^24,47^ may explain the longer latency of small saccades and why very nearby targets often fail to elicit them. For a microsaccade to be elicited, the activity in the selected location of the rostral colliculus needs to be higher than a baseline, and the activity between the two colliculi needs to be sufficiently imbalanced. Irrespective of the specific mechanisms, our results show that the probability of performing a microsaccade and its efficacy in re-centering gaze are correlated (Fig. 6), suggesting that the same neural processes affect both parameters, or, perhaps, that the oculomotor system takes into account its own limits in executing a motor plan.

The results of this study show that small saccades shift gaze with high accuracy and precision. What are the reasons for this behavior? Since vision is not uniform within the foveola^2^, the most obvious advantage offered by this degree of motor control is the capability to center a preferred retinal locus on the stimulus in high-acuity tasks. However, there may be other benefits from this behavior. For example, microsaccade preparation in itself may lead to visual enhancements, at the target location in the fovea, as it happens for larger saccades^49,50^. This latter idea is consistent with the recent finding of high-resolution attentional control within the foveola^51^. Taking advantage of these perceptual enhancements would require accurate and precise control of small saccades.

## Methods

### **Participants**

A total of 11 subjects took part in the experiments. Five emmetropic subjects participated in the main experiment of the study (average age range: 20-30 years old; 3 females). These subjects were experienced in serving as subjects for oculomotor research experiments, as they had participated in previous studies conducted in the laboratory with the same apparatus. Six other subjects (4 females; two experienced) participated in a control study with stimuli at $20^\prime$ from the fixation marker. All 11 subjects were naïve about the purposes of the study.

Informed consent was obtained from all participants following the procedures approved by the institutional review boards of the University of Rochester and the Boston University Charles River Campus. All methods were performed in accordance with the relevant guidelines and regulations of the institutional review boards of the University of Rochester and the Boston University Charles River Campus.

### **Apparatus**

Stimuli were displayed on a fast phosphor CRT monitor (Iyama HM204DT) at a vertical refresh rate of 100 Hz and spatial resolution of $1600\times 1200$ pixel. Subjects were kept at a fixed distance of 126 cm from the monitor by means of a dental-imprint bite bar and a head rest, which prevented head movements. Stimuli were observed monocularly with the right eye, while the left eye was patched. The movements of the right eye were acquired at 1 kHz by means of a Generation 6 Dual Purkinje Image (DPI) eyetracker (Fourward Technologies). This eyetracker is well-suited for the purpose of studying small saccades; its high linearity and low internal noise enable measurements of movements as small as $1^\prime$^16,34^.

Stimuli were rendered by means of EyeRIS, a custom system for flexible gaze-contingent display control^52^. This system acquires eye movement signals from the eyetracker, processes them in real-time, and updates the stimulus on the display within the interval required to render two frames on the display. Previous analysis of EyeRIS performance has shown that this system is capable of stabilizing images on the retina with precision higher than $1^\prime$^51,53^.

### **Experimental Procedures**

Data were collected via several experimental sessions. Each session started with preliminary setup operations necessary for tuning the apparatus and then continued with the execution of blocks of trials. Subjects had ample opportunity to rest within blocks, so that they were never constrained in the experimental setup for more than 10 minutes consecutively.

*Gaze localization.* Accurate localization of the line of sight on the display is essential in this study. Conversion of the eye-tracker’s output into display coordinates is normally achieved via calibration procedures in which the observer successively fixates on a grid of points. In this approach, the instability of visual fixation directly translates into uncertainty in gaze localization^17^. The resulting error can be as large as the foveola^54^, which is not sufficient for the purpose of this study.

To achieve finer gaze localization, we used a two-step gaze-contingent calibration procedure, which greatly reduces dispersion of eye position measurements. This procedure has been described in previous publications and is only briefly summarized here^2,17^. In the first phase, subjects sequentially fixated on each of the 9 points of a $3\times 3$ grid, as is customary in eyetracking experiments. In the second phase, they used a joypad to refine the calibration by correcting the position of a marker displayed on the monitor at the estimated center of gaze. This procedure improves gaze localization by approximately a factor of 3 on each axis^17^, enabling the accuracy necessary for this study.

*Saccade procedure.* As explained in Fig. 1A, the standard procedure used to study larger saccades does not work well with small saccades. In this procedure, the observer first acquires fixation and then executes a saccade toward a target^8,10,55^. The fixational offset that eye movements introduce while maintaining fixation is not negligible relative to the amplitude of small saccades and needs to be taken into account to evaluate performance. Furthermore, the retinal vector subtended by the target and the point where subjects attempt to fixate differs from the ideal saccade vector and may affect its execution.

To overcome these problems, in this study, the fixation marker moved in real-time on the display following the observer’s eye movements so to remain fixed at the very center of gaze (Fig. 1B). The saccade target was then presented at a predetermined location relative to the current gaze position. This paradigm ensured that, at the target’s appearance, the retinal and saccade vectors coincided.

Experiments were conducted in a dimly illuminated room. Subjects were instructed to fixate toward the center of the display. This is the region with optimal eye-tracking and gaze localization. They were aided in doing so by four peripheral arches ($4^\circ$ eccentricity), which remained on the monitor for the entire duration of the trial. Each trial started with the appearance of the fixation marker (a $3^\prime \times 3^\prime$ square) on a dark background. The fixation marker appeared at the currently estimated position of the center of gaze and followed the line of sight so to remain immobile on the retina. If the observer maintained fixation within a 30$^\prime$ radius region from the center, a saccade target (a $2^\prime \times 2^\prime$ square) appeared after 1 s at a desired location relative to the center of gaze. Stabilization of the fixation marker was turned off at the appearance of the saccade target, so that the marker remained fixed on the display at its latest position, a procedure that allowed for the retinal motion normally present during saccade planning. Subjects were instructed to look at the target as accurately and fast as possible and to press a button on a joypad once their gaze was centered on the target. The target’s position varied in each trial, selected from a pool of 40 different positions (Fig. 1C).

To ensure that the fixation marker was well positioned on the preferred retinal locus of fixation, we discarded all trials in which the standard deviation of movement on each axis exceeded $20^\prime$ during the fixation period. These trials were likely the outcome of not accurately centering the retinally-stabilized fixation marker on the preferred retinal locus, an operation that result in large directional pursuits. All subjects were able to maintain accurate fixation, and no noticeable individual differences were observed. For every individual subject, the dispersion of gaze (the standard deviation of gaze position) was below 8$^\prime$ on both axes, and the total number of trials in which fixational instability exceeded threshold summed up to less than 5%. Across subjects, the standard deviation of the dispersion of gaze was only $\sim 0.5^\prime$ on the horizontal axis and $\sim 1^\prime$ on the vertical axis. Thus, all subjects exhibited a similar high degree of stability during fixation. Gaze velocity during this period was on average $44\pm 15^\prime /\hbox {s}$.

In a control experiment, subjects were asked to maintain fixation on a central marker (a $2^\prime \times 2^\prime$ square) surrounded to the left and right by two squares ($5^\prime \times 5^\prime$) at an eccentricity of $20^\prime$. A central cue instructed subjects to shift their gaze in the indicated direction. Only trials in which subjects maintained gaze within $5^\prime$ of the fixation marker were included in analysis.

## Data analysis

Oculomotor traces were segmented into separate periods of ocular drifts and saccades on the basis of the speed of the trajectory, as previously described^2^. In brief, eye movements with minimal amplitude of $3^\prime$ and peak velocity higher than $3^{o}/s$ were selected as possible saccades. Consecutive events closer than 15 ms were merged together, a method which automatically excluded possible post-saccadic overshoots^56,57^. Saccade amplitude was defined as the modulus of the vector connecting the two locations at which eye speed became greater (saccade onset) and lower (saccade offset) than $3^{o}/s$. Classification of eye movements was performed automatically and then validated by human experts. All trials in which eye tracking was not continuous were discarded. Data will be made available upon reasonable request.

Saccade accuracy was measured by means of the landing error $S_E$, the vector between the landing position and the target. To examine the advantages of executing a saccade relative to those of maintaining fixation, we compared the average accuracy of saccades $\overline{S}_E$ to that of ocular drift $\overline{D}_E$. The latter was estimated over all the trials in which the target did not elicit a saccade, by taking the eye position at the moment the observer’s reported to be accurately fixating on the target via button press. The saccade gain in Fig. 6C is defined as $(\overline{S}_E-\overline{D}_E)/T$, where *T* is the target’s eccentricity at the time it appears on the display.

Saccade precision was quantified by means of the *Dispersion Index*, defined as the area within the 68^th^ percentile confidence ellipse in the distribution of landing points:

$$\begin{aligned} DI = 2k\pi \sigma _{x}\sigma _{y}\sqrt{1-\rho _{xy}^2} \end{aligned}$$

where $\sigma _x$ and $\sigma _y$ represent the standard deviation of the two Cartesian components of the saccadic landing error, and $\rho _{xy}$ is their correlation coefficient. $k = 1.14$ was selected to obtain the area within one standard deviation from the average landing position. This index has been used extensively in the literature^19,58^ and can be regarded as the direct extension of the concept of variance to two dimensions.

### **Neural modeling**

We examined whether our experimental data are compatible with the predictions of a standard model of the superior colliculus^44^. This model establishes a transformation between retinal $(R, \phi )$ and collicular (*u*, *v*) coordinates:

1 $$\begin{aligned} u&= B_u \log \left( \sqrt{R^2 + A^2 + 2AR \cos \phi }\right) - B_u \log A \end{aligned}$$

2 $$\begin{aligned} v&= B_v \arctan \left( \frac{R \sin \phi }{ A + R \cos \phi } \right) \end{aligned}$$

where $B_u$ and $B_v$ are scalars that determine, respectively, the horizontal and vertical resolution of the colliculus map, and *A* controls the gain of the transformation. These parameters were constrained by recent neurophysiological data from the rostral colliculus^46^. As in VanGisbergen et al^45^, we assumed a Gaussian profile of neural activity with mean centered at the saccade target location and independent standard deviations on the radial ($\sigma _R$) and orthogonal ($\sigma _\perp$) axes. These deviations were allowed to vary linearly with eccentricity, with slopes $m_R$ and $m_\perp$ on the two axes. Collicular response fields were transformed into distributions of saccade landing positions in retinal space, as previously described in the literature^45^.

The data in Fig. 7 are predictions obtained after optimizing model parameters via nonlinear least squares. The optimal parameters for both the horizontal and vertical data were $A=1.1$, $B_u=1.7$, $B_v=1.8$, $\sigma _R = 0.08$, $\sigma _\perp = 0.11$, $m_R = -0.02$, and $m_\perp = -0.03$.
